# The short-term economic burden of gestational diabetes mellitus in Italy

**DOI:** 10.1186/s12884-018-1689-1

**Published:** 2018-02-23

**Authors:** Michela Meregaglia, Livia Dainelli, Helen Banks, Chiara Benedetto, Patrick Detzel, Giovanni Fattore

**Affiliations:** 1 CERGAS (Centre for Research on Health and Social Care Management), Bocconi University, Via Roentgen 1, 20136 Milan, Italy; 2 Nestlé Research Center, Nestec SA, Route du Jorat 57, 1000 Lausanne, Switzerland; 3 Department of Gynecology & Obstetrics, S. Anna Hospital, University of Turin, Via Ventimiglia 3, 10126 Turin, Italy; 4 Department of Policy Analysis and Public Management, Bocconi University, Via Roentgen 1, 20136 Milan, Italy

**Keywords:** Gestational diabetes mellitus (GDM), Hospital discharge database (HDD), Delivery outcomes, Costs, Italy

## Abstract

**Background:**

The incidence of Gestational Diabetes Mellitus (GDM) is rising in all developed countries. This study aimed at assessing the short-term economic burden of GDM from the Italian healthcare system perspective.

**Methods:**

A model was built over the last pregnancy trimester (i.e., from the 28th gestational week until childbirth included). The National Hospital Discharge Database (2014) was accessed to estimate delivery outcome probabilities and inpatient costs in GDM and normal pregnancies (i.e., euglycemia). International Classification of Disease-9th Revision-Clinical Modification (ICD9-CM) diagnostic codes and Diagnosis-Related Group (DRG) codes were used to identify GDM cases and different types of delivery (i.e., vaginal or cesarean) within the database. Neonatal outcomes probabilities were estimated from the literature and included macrosomia, hypoglycemia, hyperbilirubinemia, shoulder dystocia, respiratory distress, and brachial plexus injury. Additional data sources such as regional documents, official price and tariff lists, national statistics and expert opinion were used to populate the model. The average cost per case was calculated at national level to estimate the annual economic burden of GDM. One-way sensitivity analyses and Monte Carlo simulations were performed to quantify the uncertainty around base case results.

**Results:**

The amount of pregnancies complicated by GDM in Italy was assessed at 54,783 in 2014 using a prevalence rate of 10.9%. The antenatal outpatient cost per case was estimated at €43.7 in normal pregnancies compared to €370.6 in GDM patients, which is equivalent to a weighted sum of insulin- (14%; €1034.6) and diet- (86%; €262.5) treated women’s costs. Inpatient delivery costs were assessed at €1601.6 and €1150.3 for euglycemic women and their infants, and at €1835.0 and €1407.7 for GDM women and their infants, respectively. Thus, the overall cost per case difference between GDM and normal pregnancies was equal to €817.8 (+ 29.2%), resulting in an economic burden of about €44.8 million in 2014 at national level. Probabilistic sensitivity analysis yielded a cost per case difference ranging between €464.9 and €1164.8 in 80% of simulations.

**Conclusions:**

The economic burden of GDM in Italy is substantial even accounting for short-term medical costs only. Future research also addressing long-term consequences from a broader societal perspective is recommended.

## Background

Gestational Diabetes Mellitus (GDM) is defined as “any degree of glucose intolerance with onset or first recognition during pregnancy” and represents one of the most common complications [1]. GDM is associated with higher risk for adverse pregnancy outcomes as well as an increased probability of developing future type 2 diabetes mellitus in both mother and infant [2–5].

In Italy, a 75-gr oral glucose tolerance test (OGTT) for GDM is recommended at weeks 16–18 and/or at week 24–28 of pregnancy according to pre-defined risk factors including age, body mass index (BMI), family history of type 2 diabetes, previous history of GDM, and ethnicity [6]. According to the criteria promoted by the International Association of Diabetes and Pregnancy Study Groups (IADPSG) in 2010 [7], GDM prevalence was recently assessed at around 11% in Italy [8, 9]. This rate is 25% higher than the value estimated ten years before with the old criteria and may affect both the pre- and post-natal healthcare burden in the near future [8–12]. The prevalence rate is aligned with data from other countries, where GDM is estimated to affect between 1% and 15% of pregnancies depending on ethnicity and diagnostic criteria adopted [12]. The aim of this study was to estimate the economic burden of GDM over the last pregnancy tem in Italy compared to normal pregnancies (i.e., euglycemia) from a national healthcare system perspective.

## Methods

A model covering a period of three months (around 90 days) ranging from the 28th gestational week until childbirth (included) was built in Microsoft Excel (2013) and Tree Age Pro (2015).

### Data sources

Data from the Italian National Hospital Discharge Database (HDD) provided by the Ministry of Health were accessed in order to obtain information about the women’s characteristics and types of childbirth in GDM and normal pregnancies [13]. The database covers all hospitalizations in any public or private healthcare facility in any part of Italy. Women who gave birth were identified through specific Diagnosis-Related Group (DRG) codes (370–375) for birth or using International Classification of Disease-9th Revision-Clinical Modification (ICD9-CM) diagnostic codes that indicated childbirth (details are available on request) where resulting DRG codes were outside the expected range (e.g., for severe complications requiring surgery). GDM cases were discerned in the HDD using the ICD9-CM code 648.8, either in the birth admission or through linking the identification (ID) number of the mother to admissions during a three-month period prior to delivery. DRG codes (370–375) were used to discriminate among the different delivery methods and calculate the corresponding probabilities and costs in GDM and euglycemia (excluding those admissions for childbirth with other DRG codes). A separate database of the infants’ hospitalizations for birth was extracted, which was used to estimate the average hospitalization costs of infants affected by neonatal complications frequently associated with GDM as compared to a normal newborn’s cost.

Probabilities of GDM-related neonatal outcomes were obtained from the literature since it was not possible to identify children born to GDM mothers. In HDD, about one third of the newborns lack a unique ID code (i.e., unique IDs, assigned after birth, are not always included in the birth discharge record); the date of birth, age in days and residence are not provided; and no code exists to link infants with their mothers in the dataset. After a review of the literature, we agreed to rely on a large retrospective study of 36,241 women with and without GDM that allowed all risk varying according to the presence or not of macrosomia (i.e., birthweight ≥4 kg) [14]. Indeed, GDM is an established risk factor for macrosomia, which is in turn associated with several adverse neonatal outcomes [2]. The probability of giving birth to a normal infant was obtained by difference in the model. The number of births in 2014 (502,596) as reported by official statistics [15] was used as a ‘proxy’ of the number of pregnant women in the same period, although their number might be slightly different due to multiple pregnancies and stillbirths.

### Cost analysis

The cost analysis was conducted from the perspective of the Italian National Health Service. Italy has a tax-based system funded mainly through reimbursement tariffs using DRGs for hospital care and national/regional tariffs for outpatient services and materials (with some use of additional subsidies from individual regions to their hospitals and patient co-payments for medications and outpatient services). In this study, antenatal outpatient and inpatient delivery costs broken down between mother and infant were calculated in GDM and euglycemia, respectively; DRG tariffs were used as a ‘proxy’ for inpatient costs. No discounting was applied as the time horizon was shorter than one year.

Mothers’ delivery costs were calculated as DRG tariffs adjusted for the length of stay (LOS). In detail, a cost per admission day was obtained for each delivery method by dividing the official tariff by the average LOS in days across all women in labor identified in the HDD. Then, the daily cost was multiplied by the average LOS in GDM and euglycemic women, respectively, in order to obtain the average cost of a delivery stay in the two women’s groups. In this cost calculation, we excluded ‘outliers’ defined as admissions with a LOS above the 99° percentile in the HDD.

From the infant’s perspective, no cost values were assigned to macrosomia as such, but only to the events that followed; we calculated the average cost of admissions reporting each complication considered in the model adjusted for the LOS. For example, the cost associated with respiratory distress represented the average reimbursement tariff of all admissions reporting ICD9-CM diagnostic code 769 (neonatal respiratory distress) in the HDD 2014. In the model, the cost of a normal childbirth was assessed at €560 (DRG 391).

An average cost per case was obtained in GDM and euglycemia, respectively, as the sum of the products of probabilities and costs attached to each maternal and neonatal event considered in the model. Therefore, the annual economic burden of GDM in Italy for the year 2014 was estimated by multiplying the number of GDM cases (using a prevalence rate of 10.9% [8]) by the difference in cost per case between GDM and euglycemia [2].

### Sensitivity analyses

We conducted both deterministic and probabilistic sensitivity analyses in order to test the robustness of base case results. One-way sensitivity analyses were applied by varying (± 20%) the probabilities retrieved from the literature (i.e., infant complications) and all inpatient costs in the model. In probabilistic sensitivity analysis (i.e., Monte Carlo simulations), probabilities were modeled using a beta distribution and costs using a gamma distribution.

## Results

### Mother’s characteristics

After exclusions for lack of a unique (blinded) ID (44,402, 9.1%) and accounting for multiple pregnancies in one year (122), the characteristics of the 445,812 pregnant women uniquely identified in the database and delivering in 2014 were preliminarily investigated and presented in Table 1. Women with a GDM diagnosis were more likely to be in advanced maternal age, overweight - BMI ≥25 Kg/m^2^ and/or obese - BMI ≥30 Kg/m^2^ (ICD9-CM: 649.1; 278.0), have a nationality other than Italian and a lower educational level compared to euglycemic ones.

**Table 1:** Mother’s characteristics by euglycemic status

	GDM (*N* = 11,540)		Euglycemia (*N* = 434,272)		Source
Age (mean ± SD)	33.7 ± 5.4		31.6 ± 5.6		[13]
Advanced age (≥35)	5369	46.5%	140,238	32.3%	[13]
Overweight/Obesity	276	2.4%	1190	0.3%	[13]
Foreign citizenship	3920	34.0%	82,433	19.0%	[13]
University degree^a^	1370	13.3%	61,349	15.1%	[13]

^a^Education level was reported in 416,079 of records (93.3%)

### Antenatal outpatient costs

**Fig. 1:**
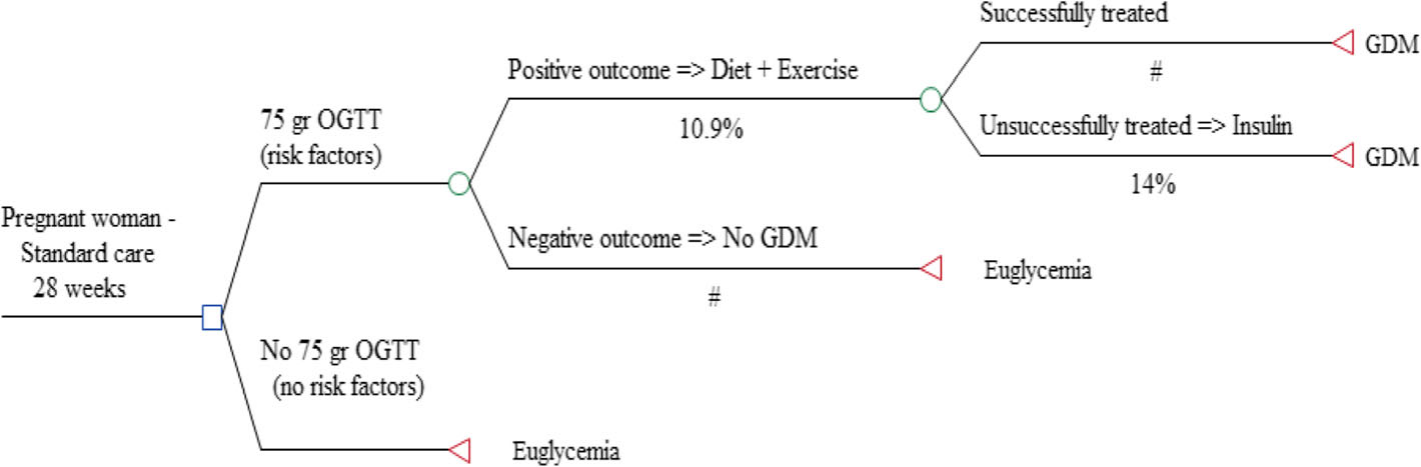
Screening and treatment path for GDM. The figure shows the generic framework of GDM screening path as recommended by Italian guidelines. The square node on the far left symbolizes the choice between the two screening options; circles represent chance events. The symbol # indicates that probabilities of that branch are complementary to those of the parallel branch. Triangles represent the terminal nodes

The screening and treatment path for GDM, as recommended by Italian guidelines, is reported in Fig. 1 [6]. In this study, we assumed all GDM diagnoses occurred at the 28th week of gestation. Accordingly, Table 2 summarizes resources consumption and costs associated with the different blood glucose states during the last pregnancy trimester. Insulin-treated GDM patients (14%) [16] perform a blood glucose test four times a day (i.e., fasting, and 1 h after breakfast, lunch, and dinner) with a portable glucometer, while those under a special diet and exercise regimen only can limit testing to two surveys per day [10, 17]. In case of variable consumption frequencies (e.g., once or twice a day), the lowest bound (e.g., once a day) was conservatively adopted to estimate the costs. The cost of insulin therapy was assessed by referring to the most prescribed drug (i.e., insulin aspart) for GDM in Italy. Additional visits and exams, including a visual field test for eye complications, were reported in GDM women during pregnancy [17]. The cost of one oral 75-g OGTT was included to account for an extra test prescribed to GDM women shortly after delivery [6].

**Table 2:** Antenatal outpatient costs by mother’s euglycemic status

Category	Resource	Consumption			Code	Unit cost (€)	Total costs per case (€)			Source
		*GDM (Diet)*	*GDM (Insulin)*	*Euglycemia*			*GDM (Diet)*	*GDM (Insulin)*	*Euglycemia*	
Screening	OGTT 75 g	2	2	1	90.26.4	2.38	4.76	4.76	2.38	[6]
Self-monitoring blood glucose kit	Glucometer	Yes	Yes	No	–	Free of charge	0.0	0.0	0.0	Expert opinion
	Test strip	2/day	4/day	No	–	0.55	99.0	198.0	0.0	[10, 17, 23]
	Finger stick	2/day	4/day	No	–	0.10	18.0	36.0	0.0	[24]
Insulin therapy	Insulin (shot)	No	1–2 shots/day	No	–	6.34	0.0	570.6	0.0	[17, 25]
	Needle	No		No	–	0.14	0.0	12.6	0.0	[24]
	Syringe	No		No	–	0.11	0.0	9.9	0.0	[24]
Visits and exams	Obstetric visit	Every 3–4 weeks	Every 1–2 weeks	Every 5–6 weeks	89.26	20.66	62.0	124.0	41.3	Expert opinion; [26]
	Obstetric ultrasound	2 (at 32th week and 36th week)		No	88.78.2	30.99	62.0	62.0	0.0	Expert opinion; [17]
	Visual field test	1		No	95.05	16.78	16.8	16.8	0.0	[17]
Total costs							262.5	1034.6	43.7	

### Inpatient costs

Maternal events resulting in hospitalization in the model represented the different delivery options as described by the DRG system (codes: 370–375); the corresponding probabilities were retrieved from the HDD for normal pregnancies and those complicated by GDM, respectively (Table 3). The analysis was performed on 442,285 deliveries after excluding 3650 records with other DRG codes.

**Table 3:** Delivery options probabilities by mother’s euglycemic status

DRG	Description	Euglycemia (*N* = 430,851)		GDM (N = 11,434)		Source
370	Caesarean section with CC	11,437	2.6%	538	4.7%	[13]
371	Caesarean section without CC	145,212	33.7%	4019	35.2%	[13]
372	Vaginal delivery with CC	9447	2.2%	447	3.9%	[13]
373	Vaginal delivery without CC	260,036	60.3%	6287	55.0%	[13]
374	Vaginal delivery with sterilization and/or dilatation and curettage	4274	1.0%	125	1.1%	[13]
375	Vaginal delivery with other interventions but sterilization and/or dilatation and curettage	445	0.1%	18	0.2%	[13]

The health events considered for infants were hypoglycemia (blood glucose < 35 mg/dL), hyperbilirubinemia (total serum bilirubin > 5 mg/dL), respiratory distress, shoulder dystocia and brachial plexus injury as reported by the study from Esakoff et al. [14]; the risk of macrosomia was retrieved instead by an Italian study [18] (Table 4).

**Table 4:** Neonatal events probabilities by mother’s euglycemic status

	Macrosomia	Hypoglycemia	Hyperbilirubinemia	Shoulder dystocia	Respiratory distress	Brachial plexus injury	Normal newborn
Euglycemia	Yes (7.4%)	2.4%	7.6%	6.0%	1.7%	0.7%	81.6%
	No	1.2%	9.1%	0.9%	1.2%	0.1%	87.5%
GDM	Yes (8.7%)	5.3%	13.2%	10.5%	4.0%	2.6%	64.4%
	No	2.6%	10.4%	1.6%	1.5%	0.2%	83.7%
Source	[18]	[14]	[14]	[14]	[14]	[14]	Own calculation

Inpatient costs estimated for each event considered in the model are reported in Table 5. For neonatal complications, a unique cost value was reported since the database does not allow to distinguish between children born from GDM and euglycemic mothers.

**Table 5:** Inpatient costs (€) of mother’s and neonatal events

	Code(s)	Event	Cost (€)		Source
			Euglycemia	GDM	
Mother	370^a^	Caesarean section with CC	2774.5	2938.5	[13]
	371^a^	Caesarean section without CC	2084.3	2373.0	[13]
	372^a^	Vaginal delivery with CC	1610.3	1802.8	[13]
	373^a^	Vaginal delivery without CC	1269.3	1385.2	[13]
	374^a^	Vaginal delivery with sterilization and/or dilatation and curettage	2116.0	2330.9	[13]
	375^a^	Vaginal delivery with other interventions but sterilization and/or dilatation and curettage	2842.0	3265.9	[13]
Infant	769^b^	Respiratory distress	24,337.8		[13]
	775.0/775.6^b^	Hypoglycemia	6571.8		[13]
	774.2/774.6^b^	Hyperbilirubinemia	2854.2		[13]
	767.6^b^	Brachial plexus injury	1671.9		[13]
	660.4^b^	Shoulder dystocia	1407.6		[13]
	391^a^	Normal newborn	560.0		[13]

^a^DRG; ^b^ICD9-CM

### Overall costs

As reported in Table 6, the base case analysis yielded a cost per GDM case equal to €3613.4 divided into antenatal outpatient (€370.6) and inpatient costs for mother (€1835.0) and infant (€1407.7).

**Table 6:** Mother’s and infant’s cost per case (€) by mother’s euglycemic status

	Outpatient	Inpatient		Inpatient cost/case	Total cost/case
	Mother	Mother	Infant		
Euglycemia	43.7	1601.6	1150.3	2751.9	2795.6
GDM	370.6	1835.0	1407.7	3242.8	3613.4
Diet (86%)	262.5	–	–	–	–
Insulin (14%)	1034.6	–	–	–	–
Delta	326.9	233.4	257.4	490.9	817.8

GDM outpatient costs corresponded to a weighted sum of insulin- and diet-treated women’s costs and were on average €326.9 higher than in euglycemic women. Similarly, total inpatient costs (mother and infant) in GDM (€3242.8) outweighed those in normal pregnancies (€2751.9). Overall, each pregnancy affected by GDM cost 29.2% more than a pregnancy without GDM, with a cost per case difference of €817.8. The number of pregnancies affected by GDM was estimated at 54,783 (out of 502,596 births) in 2014, resulting in an extra cost of around €44.8 million for the Italian National Health Service.

### Sensitivity analyses

In Table 7 we showed the effects of varying selected parameters on the inpatient cost per case difference between GDM and euglycemia. The largest impact on Δ cost/case was obtained by varying ±20% the cost of cesarean section without complications in GDM, which yielded an interval of the cost difference equal to €324.1 - €657.7.

**Table 7:** One-way sensitivity analysis on the inpatient costs per case (€)

	Cost/case (euglycemia)	Cost/case (GDM)	∆ cost/case
Base case	2751.8	3242.7	490.9
*Costs (€)*			
Hypoglycemia			
−20%	2734.9	3205.4	470.5
+ 20%	2768.8	3280.0	511.2
Respiratory distress			
− 20%	2691.6	3159.1	467.5
+ 20%	2812.1	3326.3	514.2
Cesarean section with CC (GDM)			
− 20%	2751.8	3215.0	463.2
+ 20%	2751.8	3270.4	518.6
Cesarean section without CC (GDM)			
− 20%	2751.8	3075.9	324.1
+ 20%	2751.8	3409.5	657.7
Vaginal delivery without CC (GDM)			
− 20%	2751.8	3090.3	338.5
+ 20%	2751.8	3395.0	643.2
Cesarean section without CC (euglycemia)			
− 20%	2611.4	3242.7	631.3
+ 20%	2892.3	3242.7	350.4
Vaginal delivery without CC (euglycemia)			
− 20%	2598.6	3242.7	644.1
+ 20%	2905.1	3242.7	337.6
*Probabilities*			
Hyperbilirubinemia (GDM/no macrosomia)			
− 20%	2751.8	3199.1	447.3
+ 20%	2751.8	3286.3	534.5
Hypoglycemia (GDM/no macrosomia)			
− 20%	2751.8	3214.2	462.4
+ 20%	2751.8	3271.2	519.4
Respiratory distress (GDM/no macrosomia)			
− 20%	2751.8	3177.6	425.8
+ 20%	2751.8	3307.8	556.0
Hyperbilirubinemia (euglycemia/no macrosomia)			
− 20%	2713.2	3242.7	529.5
+ 20%	2790.5	3242.7	452.2
Respiratory distress (euglycemia/no macrosomia)			
− 20%	2699.0	3242.7	543.7
+ 20%	2804.7	3242.7	438.0

**Fig. 2:**
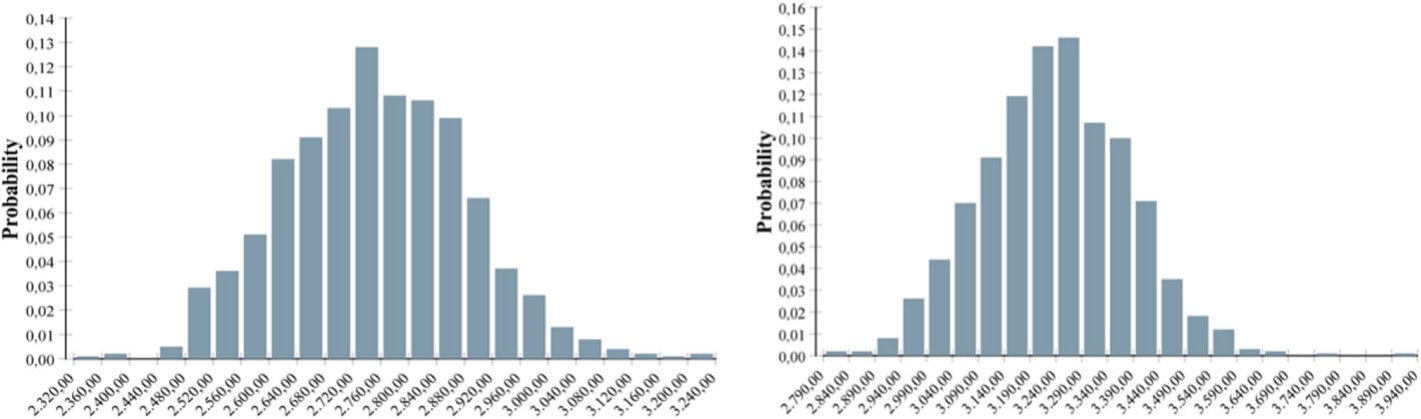
Monte Carlo probability distributions in euglycemia (left) and GDM (right). The figure shows Monte Carlo probability distribution (1000 iterations) of the expected inpatient cost per case in euglycemia (left) and GDM (right). The most likely value (the highest bar in the histogram) corresponds to the base case result

Figure 2 displays Monte Carlo probability distributions (1000 iterations) of the expected inpatient cost per case in euglycemic and GDM women, respectively. The possible values ranged between €2326.6 and €3206.6 in euglycemia and €2792.0 and €3890.4 in GDM. However, 80% of the expected cost values were included in the range €2577.7 - €2915.7 in euglycemia and €3053.7 - €3415.6 in GDM. Under these simulations, inpatient cost per case difference between GDM and euglycemia might vary between €138.0 and €837.9 which, summed with the outpatient cost/case difference (€326.9), would give an overall cost/case difference range of €464.9 - €1164.8.

## Discussion

### Main findings

There is a lack of research on the burden associated with GDM in many countries, masking its potential importance for policy-makers. To our knowledge, this is the first study to estimate the economic consequences of GDM in Italy.

This analysis showed that pregnant women affected by GDM are more likely to present established risk factors such as obesity, advanced maternal age, non-Italian citizenship, and low educational level and to experience adverse pregnancy outcomes. Even accounting for short-term medical costs only, the yearly economic burden of GDM was substantial (€44.8 million) in 2014. At the outpatient level, a relevant difference in cost (+ 748%) was observed between GDM and euglycemic women, while in terms of hospitalization, mother and infant costs were respectively 14.6% and 22.3% higher than in euglycemia. These results remained robust after performing both deterministic and probabilistic sensitivity analysis.

### Strengths and limitations

The main strength of this study was the availability of administrative data on hospital discharges for all women who delivered in 2014 in Italy. As a check on the reliability of the data, we measured an infant mortality rate of 3.3 per 1000 live births, which is in line with the rate of 3.1 in published data for Italy [15]. However, our database contained fewer women (445,812) uniquely identified as giving births than those reported by official statistics (502,596), likely due to exclusions for incomplete (blinded) ID codes and inconclusive diagnostic and DRG coding. We also found wide, unexplained variation in the percentage of women with a GDM diagnosis among regions (from 0.5% in Aosta Valley to 6.1% in Tuscany) and a low overall GDM rate (< 3%) in hospital delivery records, even though clinical studies [8, 9] and expert opinion estimated the prevalence to be 10% or more in Italy. This inconsistency (and presumed under-reporting) is likely due to variation among regions in coding practices and number of diagnostic code fields reported at discharge, plus a lack of financial incentive to consistently record secondary diagnoses. This is a main limiting factor in using administrative data rather than medical records for research purposes.

This analysis also presented a few additional limitations. First, the costs associated to GDM were likely to be underestimated for several reasons. For example, we only considered the public expenditure for GDM, while out-of-pocket expenses for extra visits and exams, nutritional supplements, food substitution and physical activity may be substantial. Moreover, we did not consider that GDM can be diagnosed at an earlier pregnancy stage (16th–18th week) in high-risk women, nor that it may be under diagnosed in women without any of the risk factors required to perform the OGTT test. We also disregarded that women can be admitted at the hospital during the pre-delivery period for complications related to GDM, and that patient compliance in performing daily glucose tests or assuming the prescribed therapies may not be perfect.

Second, the data sources for mother’s and infant’s events were very different, since for the latter it was not possible to derive GDM-related probabilities from the HDD due to the unknown mother’s glycemic status; in particular, we referred to a single study [14] reporting perinatal outcomes in infants born in San Francisco before 2006. Until now, no study has reported all the neonatal outcomes of interest for Italy through a comparison between GDM and non-GDM women, thus it was not possible to refer to a more recent and country-specific study. However, some of the outcomes reported by the reviewed study [14] were aligned with the values indicated in the Italian literature; for example, in GDM, fetal distress was estimated at 3.4% and hypoglycemia at 2.5% in the study by Lapolla et al. [18]; unfortunately, the authors did not provide these figures for normal pregnancies.

Third, we adopted a national reference for reimbursement tariffs to model costs, although tariffs may vary at the regional level in Italy. The inter-regional unitary tariff for DRGs is the best available estimate of hospitalization costs across regions, although it represents an approximation of real costs; the production cost, indeed, is only one of the variables used for setting tariffs [19]. Thus, we performed sensitivity analysis on inpatient costs to consider any potential variations.

### Interpretation

According to the literature, the GDM rate is growing rapidly worldwide with a consequent large healthcare cost increase [20]. A cluster-randomized trial from Finland [21] reported that mean healthcare costs, including both inpatient and outpatient care before and after delivery, were 25% higher in women diagnosed with GDM (€6432) than among euglycemic women (€5143) in the 2007–2009 period. Moreover, women affected by GDM were more likely to be in advanced maternal age, overweight or obese and with a low educational level.

A recent modeling study [2] conducted in the US estimated a short-term cost of GDM of $1.8 billion in 2014 (corresponding to around €1.5 billion) using a prevalence rate of 5.5%, which might underestimate the real GDM burden since the US did not adopt the IADSPG criteria [22]. The average additional cost per case was $15,593 (around €13,700).

Due to differences in methodological approaches and healthcare financing systems, findings from previous studies were hardly comparable with ours; however, they confirmed a substantial economic burden of GDM at least in developed countries. This study, unlike the Finnish one, did not adopt an experimental design, and relied instead on heterogeneous data sources to estimate the costs of GDM in Italy; however, by using administrative data, we could review a much larger sample of observations that those generally available in empirical studies.

## Conclusions

This study should be interpreted as a first step towards further research in diabetes in pregnancy and highlights the need of collecting additional data regarding GDM in Italy and elsewhere. Parameters included in this model are likely to be used in future cost-effectiveness analyses of novel GDM treatments or preventive interventions targeted to women of childbearing age. Our analysis focused on the gestational period only, but long-term consequences of GDM in mother (i.e., type 2 diabetes) and infant (i.e., type 2 diabetes, congenital malformations, obesity, and cardiovascular diseases) are likely to increase the estimated economic burden. Thus, additional studies investigating GDM-related effects over mothers’ and infants’ lifetimes are also needed.

## Abbreviations

BMI: Body mass index
DRG: Diagnosis-Related Group
GDM: Gestational Diabetes Mellitus
HDD: Hospital Discharge Database
IADPSG: International Association of Diabetes and Pregnancy Study Groups
ICD9-CM: International Classification of Disease-9th Revision-Clinical Modification
ID: (unique) identifier
OGTT: oral glucose tolerance test
